# Differential Expression of MicroRNAs in Tumors from Chronically Inflamed or Genetic (APC^Min/+^) Models of Colon Cancer

**DOI:** 10.1371/journal.pone.0018501

**Published:** 2011-04-12

**Authors:** Brian M. Necela, Jennifer M. Carr, Yan W. Asmann, E. Aubrey Thompson

**Affiliations:** 1 Department of Cancer Biology, Mayo Clinic Comprehensive Cancer Center, Jacksonville, Florida, United States of America; 2 Department of Biomedical Statistics and Informatics, Mayo Clinic College of Medicine, Rochester, Minnesota, United States of America; Instituto Nacional de Câncer, Brazil

## Abstract

**Background:**

Chronic inflammation associated with ulcerative colitis predisposes individuals to increased colon cancer risk. The aim of these studies was to identify microRNAs that are aberrantly regulated during inflammation and may participate in transformation of colonic epithelial cells in the inflammatory setting.

**Methodology/Principal Findings:**

We have use quantitative PCR arrays to compare microRNA (miRNA) expression in tumors and control colonic epithelial cells isolated from distal colons of chronically inflamed mice and APC^Min/+^ mice. Rank order statistics was utilized to identify differentially regulated miRNAs in tumors that arose due to chronic inflammation and/or to germline APC mutation. Eight high priority miRNAs were identified: miR-215, miR-137, miR-708, miR-31, and miR-135b were differentially expressed in APC tumors and miR-215, miR-133a, miR-467d, miR-218, miR-708, miR-31, and miR-135b in colitis-associated tumors. Four of these (miR-215, miR-708, miR-31, and miR-135b) were common to both tumors types, and dysregulation of these miRNAs was confirmed in an independent sample set. Target prediction and pathway analysis suggests that these microRNAs, in the aggregate, regulate signaling pathways related to MAPK, PI3K, WNT, and TGF-β, all of which are known to be involved in transformation.

**Conclusions/Significance:**

We conclude that these four miRNAs are dysregulated at some very early stage in transformation of colonic epithelial cells. This response is not dependent on the mechanism of initiation of transformation (inflammation versus germline mutation), suggesting that the miRNAs that we have identified are likely to regulate critical signaling pathways that are central to early events in transformation of colonic epithelial cells.

## Introduction

MicroRNAs (miRNAs) are a class of small noncoding RNAs of 19 to 22 nucleotides implicated in a number of important cellular processes such as development, differentiation, proliferation, cell cycle progression, apoptosis, inflammation, and stress responses [1]–[8]. MicroRNAs are generally believed to function by binding the 3′UTR of target mRNAs and either inhibiting translation or in some cases inducing mRNA degradation [3], [9]. Bioinformatic studies suggest that miRNAs may regulate one-third of the transcriptome [10], [11]. Given their propensity to regulate numerous processes and target genes, it is no surprise that aberrant expression of miRNAs has been linked to numerous pathological conditions such as asthma, diabetes, kidney, neurodegenerative, and cardiovascular disease. In particular, differential miRNA expression has been implicated in many cancers including breast, thyroid, lung, pancreatic, and colon cancer.

To date, over a dozen studies have reported an association of aberrant miRNA expression in colon cancer [12]–[31]. Expression of miRNAs in colon tumors can be influenced by numerous clinicopathologic variables such as tumor grade and location, mutation status (p53, APC, MSI) [32]–[36], and cellular content (*i.e.* inflammatory cells) as well as pre-analytical and analytical variables such extraction method, fixation, and choice of analytical platform (sequence, qPCR, or microarray). As a result of these variables, there is no consensus concerning which miRNAs are differentially expressed in colon tumors. Nevertheless, a few microRNAs have consistently emerged as being dysregulated in colon cancer. Among these, miR-31 is consistently upregulated [12]–[17], [20], [22], [23], [25] and microRNA clusters miR-143/-145 [18], [21]–[23], [37]–[40] and miR-194/-215 [18], [21]–[23], [37], [38], [41] downregulated in colon cancer. MicroRNA −31 has been linked to cell migration and invasion in colon cancer cells [42], [43]. MicroRNAs −192 and −215 inhibit cell proliferation and induce cell cycle arrest in a p53-dependent manner [37], [44], [45]. Likewise, miR-143 and miR-145 inhibit cell growth, with this action, in part, attributed to through inhibition of target genes such as DNMT3A, IRS-1, YES1, STAT1, and FLI1 [21], [46]–[50].

One clinical variable that has not been adequately addressed is the inflammatory status of the colon tumor. A direct link between inflammation and cancer has been firmly established, with NFκB emerging as a key player [51], [52]. Inflammation is believed not only to alter and promote the tumor microenvironment, but itself, can lead directly to tumorigenesis. Patients with ulcerative colitis, a chronic, relapsing inflammation of the colon, have increased risk of developing colon cancer. A recent meta-analysis revealed probabilities of colon cancer in ulcerative colitis of 2% after 10 years, 8% after 20 years, and 18% after 30 years of disease [53]. One possible mechanism by which inflammatory pathways may influence transformation is through deregulation of miRNAs. Increasing evidence suggest that miRNAs are capable of regulating inflammatory processes and are dysregulated in various inflammatory diseases [54]. Recently, dysregulation of microRNAs has been observed in patients with ulcerative colitis [55], [56]. However, little is known about the functional consequence of dysregulation of miRNAs during chronic colitis in epithelial cells, and even less on tumorigenesis. Although limited, these studies do provide the precident that deregulation of a subset of microRNAs during chronic colitis may be associated with neoplastic and metaplastic metaplastic transformation. To this end, microRNAs may be useful biomarkers to help predict risk for malignancy in patients with long standing active disease.

The studies described below were undertaken to begin to test the hypothesis that a subset of microRNAs is dysregulated in the intestinal epithelium during colitis and contribute to carcinogenesis. We measured miRNAs by quantitative PCR low density TaqMan arrays using RNA extracted from colonic epithelial cells of mice induced with acute dextran sulfate colitis (AC), chronic colitis (CC), colitis-associated colon tumors (CAC), and colon tumors in APC^Min/+^ mice. We identified differential expression of miRNAs common and unique to each disease condition. In particular, we demonstrate that a subset of miRNAs is dysregulated in both tumors of both inflammatory and genetic origin. These miRNAs have the potential to control both anti-oncogenic and pro-oncogenic transcriptional networks, thereby influencing tumorigenic outcome.

## Results

### Comparison of analytical models using acute colitis (AC) samples

Quantification of miRNA expression in tissue samples is complicated by the fact that one has no obvious direct means to identify appropriate endogenous controls that may be used to normalize expression data and correct for differences in the amount of RNA analyzed or the efficiency of miRNA extraction or cDNA conversion in samples from different tissues. This potential problem is particularly acute in the studies such as those that will be described below in which we undertake to compare miRNA abundance in tissues derived from mice of different ages, diets, inflammatory status, and tumor burden. We therefore carried out a pilot experiment to compare different analytical tools and statistical models to identify differentially regulated miRNAs in colonic epithelial cells. To this end we extracted miRNAs from epithelial cell preparations from distal colons of control C57BL/6J mice and mice that had been exposed to 3.5% DSS in drinking water for four days. Histologically, the colons from control and DSS-treated mice were indistinguishable, with no evidence of barrier breakdown, crypt loss, or invasion by immune cells. Although these tissues formally represent a pre-inflamed state, the samples will be designated AC (acute colitis) in the follow discussion, whereas the controls will be identified as Con.

We used AB TaqMan arrays to measure the abundance of 384 miRNAs in AC and Con samples, expressed as critical thresholds (Ct) for each miRNA detector. Three analytical approaches were compared. The simplest of these was rank order statistics. Each miRNA within a given sample was force ranked on analog readout of miRNA abundance (Ct value). Detectors that were scored as ‘undetectable’ were assigned Ct values of 40, and all miRNAs with Ct = 40 were assigned the same rank within a sample. A t-test (assuming unequal variance) was then carried out to compare the rank order assignments of miRNAs in the two different sample groups so as to identify miRNAs whose mean rank order of expression changed significantly as a result of treatment. The second analytical approach involved a simple median normalization of the samples, followed by a t-test (assuming unequal variance) to compare Ct values between Con and AC samples. Differential expression is expressed as ΔCt (abbreviated DCt), which represents (meanCtMed_Norm_AC) - (meanCtMed_Norm_Con). The third method employed the Integromics Statminer package, which uses a Limma-based empirical Bayesian implementation to calculate a hyperparamter which is in turn used to modulate the variance and increase the degrees of freedom across the samples. The Statminer package also calculates variance among user-defined endogenous controls and identifies those with the least variance across all samples. The Statminer package allows multiple endogenous controls to be used to normalize the dataset. In our case the least variant endogenous controls were sno135 and miR-25, and these were used to normalize the data. Differential expression is expressed as ΔΔCt (abbreviated DDCt), which represents (meanΔCtLimma_SM_Tumor)-(meanΔCtLimma_SM_Con) Thus we compared a relatively simple model in which no attempt was made to normalize the data (rank order statistics, abbreviated Rnk_Diff), a simple median normalization model (Med_Norm), and a more complicated empirical Bayesian model that used multiple endogenous controls to normalize the data (Limma_SM).

**Figure 1:**
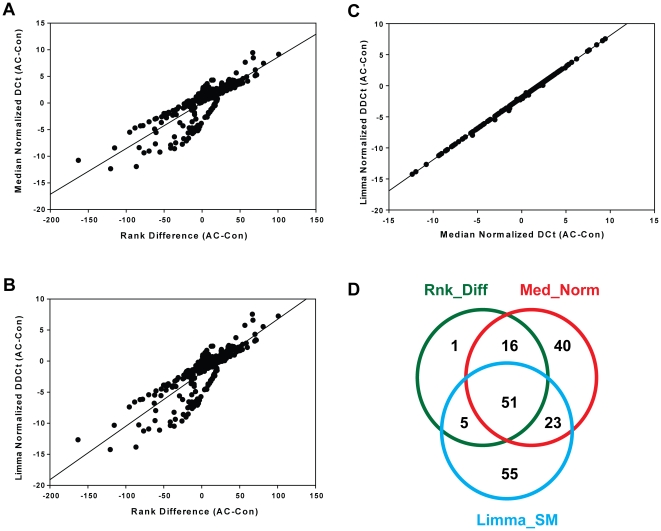
Comparison of three different normalization methods to identify differentially expressed miRNAs in DSS-induced acute colitis. Changes in rank order, defined as mean rank in AC samples minus mean rank in Con samples, are plotted against DCt values (mean Ct_AC minus mean Ct_Con) in **Panel A** or DDCt (mean DCt_AC minus mean DCt_Con) in **Panel B**. Limma normalized DDCt values for individual miRNAs are plotted against median normalized DCt values in **Panel C**. Differentially expressed miRNAs were filtered on P<0.01 and rank order change ≥10 or log2 fold change ≥1.0. Overlap between differentially expressed miRNAs identified by rank order statistics (Rnk_Diff), t-test using median normalized data (Med_Norm), or the Statminer implementation of Limma (Limma_SM) is shown in **Panel D**.

A comparison of the changes in rank order of individual miRNAs (Rank Difference AC-Con) and the log2 fold change from median normalized data [Median Normalized DCt (AC-Con)] is shown in Figure 1A, whereas a similar comparison of log2 fold change after Limma normalization to sno135 and miR-25 [DDCt(AC-Con)] is shown in Figure 1B. The distributions are obviously nearly identical, as indicated by Spearman rank correlation coefficients of 0.8324 and 0.8328 for Figures 1A and B, respectively. This similarity results from near identity of the expression data calculated from median normalized and Limma-normalized data (Figure 1C, Spearman rank correlation coefficient = 0.9996). The observation that two very different normalization protocols yielded essentially identical results probably indicates that there is very little variance, either analytical or biological, among our samples.

Using these three approaches we identified 193 miRNAs that were scored as significantly different (P≤0.01) in one or more of the analyses (listed in Table S1). We imposed additional filters of rank difference ≥(±10) and P≤0.01 to identify 73 differentially expressed miRNAs by rank order statistics. When we filtered on log2 fold change≥(±1.0) and P≤0.01 we identified 130 miRNAs that were differentially expressed in the median normalized analysis and 134 miRNAs that were differentially expressed by Limma analysis. A VENN diagram of overlap between these miRNAs is shown in Figure 1D. Somewhat to our surprise, there were only 51 miRNAs that satisfied all conditions for all models. A three dimensional correlation comparing rank difference, DCt (median normalization), and DDCt (Limma normalization) revealed a 3-dimensional correlation coefficient of 1.00 for these 51 samples (data not shown). These miRNAs represent high probability targets that are likely to play some role in the very earliest stages of inflammation. (These miRNAs are identified in bold in Table S1.) Both median normalization and Limma yielded significant numbers of outliers, whereas rank order statistical analysis exhibited almost complete overlap with at least one of the other models (Fig. 1D).

Our conclusion from this initial comparison of three different normalization methods is that they all give very similar results, in terms of relative miRNA abundance, in a dataset with little analytical or biological variance within the control and experimental groups. The different statistical models give significantly different p-values, which is probably due in large part to the small number of samples that were included in this analysis as well as the use of moderated variance (Limma) versus unmoderated variance (t-test). Overall the rank order statistics works at least as well as either of the normalized models, tends to be more conservative in terms of number of differentially regulated miRNAs, and has the additional advantages that it requires no knowledge of appropriate endogenous controls and makes no assumptions about equal amounts of RNA or equal efficiency of miRNA extraction or cDNA conversion from sample to sample. We therefore elected to proceed with our analyses using rank order statistics to identify differentially expressed miRNAs. However, changes in rank order are not informative in terms of absolute (or more precisely analog) miRNA abundance, so we relied on Limma to calculate normalized abundance, expressed as DDCt, which corresponds to −log2 fold change normalized to sno135b/miR-25.

### Identification of differentially expressed miRNAs in experimental models of colitis-associated colon cancer and familial adenomatous polyposis coli

We used AB TaqMan miRNA arrays to measure the abundance of 384 miRNAs in tumors isolated from distal colons of APC^Min/+^ mice (APC tumors) and tumors that formed in the distal colons of chronically inflamed mice (CAC tumors). The APC^Min/+^ mice were retired breeders, 120d of age, and maintained on high fat breeder's chow. CAC tumors were isolated from mice that had been subjected to 12 rounds of low level inflammation, induced by DSS as described in Materials and Methods. As control samples, we isolated epithelial cells from adjacent, uninvolved epithelium from APC^Min/+^ (APC control) or chronically inflamed (CC control) mice. Rank order statistics was used to identify candidate differentially expressed miRNAs in APC and CAC tumors. (Raw Ct values for these samples are given in Table S2.)

**Figure 2:**
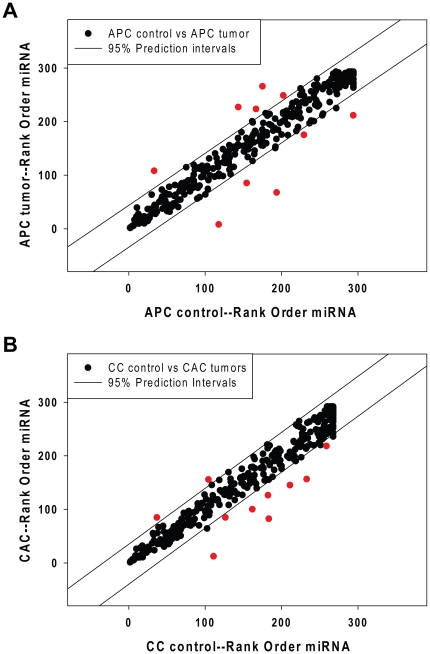
Rank order statistical analysis of differentially expressed miRNAs in tumors from APC^Min/+^ or chronically inflamed mice. Mean rank orders of miRNA abundance (based on Ct values) were determined for tumors from the distal colons or uninvolved adjacent distal colonic epithelial cells (**Panel A**). SigmaStat was used to calculate 95% confidence intervals for these data (parallel solid lines) and miRNA detectors that fell outside of these intervals are filled in red. Similarly, we compared rank order of expression of miRNAs from tumors that formed in the distal colons of chronically inflamed mice (CAC) and in uninvolved, chronically inflamed distal colonic epithelial cell preparations (CC controls), as shown in **Panel B**. MicroRNA detectors that fell outside of the 95% confidence intervals are filled in red.

The Ct values of all miRNAs in individual tumors and control samples were determined and force ranked as described above and the t-test was used to identify miRNAs whose mean rank was significantly different in controls and tumors. The average rank orders for individual miRNAs from APC control and APC tumors are displayed in Figure 2A. There was, in general, a good correspondence between rank order of miRNA abundance in APC control and APC tumor samples (Spearman Rank Order correlation coefficient = 0.973, p = 2.0E^−07^), however 10 miRNAs (shown as red symbols) fell outside the 95% prediction intervals (shown as parallel lines in Fig. 2A).

Figure 2B compares rank orders of the abundance of miRNAs measured in CCcontrol and CACtumors samples. As with the APC samples, there was a generally high correspondence between rank order abundance of miRNAs in these samples (Spearman Rank Order correlation coefficient = 0.970, p<2.0E^−07^). However, 10 miRNAs (red symbols) fell outside the 95% prediction intervals (Fig. 2B), indicating that the ranks of these miRNAs were different in controls and tumors, consistent with a high probability that these miRNAs were differentially expressed. It is our experience that very large changes in miRNA abundance (as evidenced in this case by large rank order differences) are occasionally associated with atypical kinetics of amplification of very low abundance miRNAs. We therefore visually inspected the qPCR amplification curves for each of the miRNAs in every sample and eliminated those for which log-linear amplification kinetics (1Ct/cycle) did not obtain. This curation step reduced the number of high probability differentially expressed miRNAs in APC tumors to 5 and the number of such miRNAs in CAC tumors to 7. As shown in Table 1, two miRNAs were repressed in APC tumors (miR-215 and miR-137), compared to adjacent control epithelium, whereas 3 miRNAs were induced (miR-708, miR-31, miR-135b). Only 1 miRNA was repressed in CAC tumors compared to control chronically inflamed epithelium (miR-215), whereas 6 miRNAs were induced in CAC tumors (miR-133a, miR-467d, miR-218, miR-31, miR-135b). Three miRNAs were induced in both APC and CAC samples (miR-31, miR-135b, and miR-708) and 1 miRNA was repressed in both APC and CAC samples (miR-215). In addition, 1 miRNA was uniquely repressed in APC tumors (miR-137), and 3 miRNAs were uniquely induced in CAC tumors (miR-133a, miR-467d, and miR-218). The 8 differentially expressed miRNAs in Table 1 were therefore selected for validation.

**Table 1:** Differentially expressed microRNAs in APC and CAC tumors.

	APC control vs APC tumor			CC control vs CAC tumor	
miRNA	Ave rank order diff.	p-value	miRNA	Ave rank order diff.	p-value
miR-215	−75	0.0014	miR-215	−48	0.0042
miR-137	−57	0.0206	miR-133a	38	0.0014
miR-708	69	0.0022	miR-467d	40	0.0147
miR-31	110	0.0021	miR-218	42	0.0018
miR-135b	126	<0.0001	miR-708	62	0.0014
			miR-31	98	<0.0001
			miR-135b	101	0.0014

Rank order differences were determined by subtracting the average rank order of individual microRNAs in each group of tumor samples from the rank order in the corresponding control (*e.g.* APC control rank – APC tumor rank = rank order diff.). Signficance (p-value) was assessed using the t-test (assuming unequal variance) to compare rank orders of individual microRNAs in tumors and controls.

**Figure 3:**
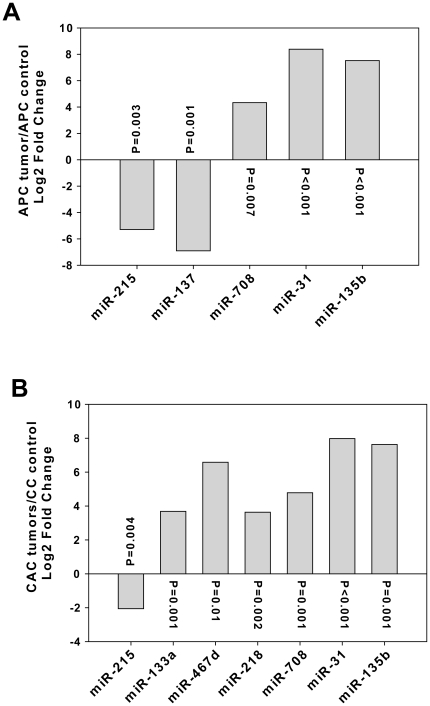
Differential expression of 8 miRNAs in tumors from APC^Min/+^ or chronically inflamed mice. MicroRNA abundance in the four different sample cohorts was normalized using Statminer and sno135/miR-25 as endogenous controls. APC controls were normalized to APC tumors (Panel A) and CC controls to CAC tumors (Panel B). The abundance of each of the 8 candidate miRNAs is represented as log2 fold change (mean Ct tumor – mean Ct control) and p-values were calculated using the Limma empirical Bayesian implementation of the moderated t-test.

Although rank order statistics is a useful tool for nominating miRNAs that are differentially expressed, a more quantitative approach is required to determine the magnitude of the response in any given comparison. To this end we used the Statminer implementation of Limma to carry out normalization and summarization of the TaqMan array expression data. The data are presented in Figure 3 as the log 2 transformation of fold change for each of the 8 focus miRNAs, where Fold Change = −[mean (DCtTumor−DCtControl)]. P-values were calculated for each comparison using the Limma empirical Bayesian implementation of the t-test to compare normalized (DCt) values for tumors and controls. As shown in Figure 3, all candidate miRNAs exhibited large, statistically significant differences in expression in APC tumors (Fig. 3A) and CAC (Fig. 3B), relative to control samples.

**Figure 4:**
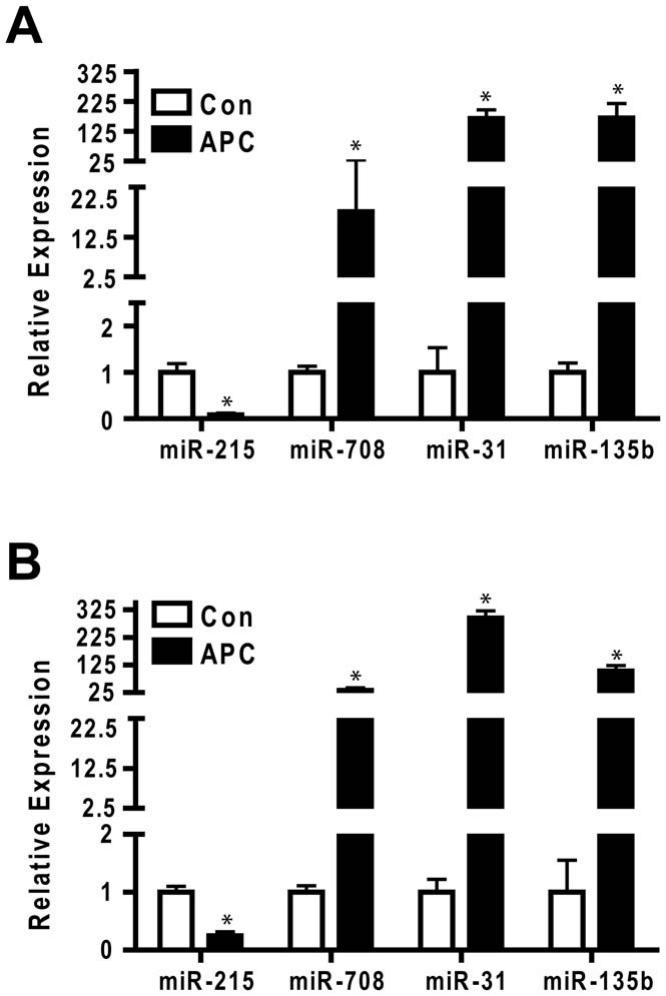
Confirmation of focus miRNAs using conventional qPCR. The RNA samples analyzed in Figures 2 and 3 were converted to cDNA using individual Taqman hairpin RT primers, as opposed to the universal primer mix used for the TaqMan arrays. Thus different cDNA preparations were prepared and assayed for miRNA abundance in APC samples (**Panel A**) and CAC samples (**Panel B**). MicroRNA abundance was normalized with sno135 and p-values calculated with the Statminer Limma protocol. Data for each miRNA were calibrated to expression (2e-DCt) in the relevant control sample. Bars represent the mean and standard deviation for each miRNA, n = 4. * indicates p-value<0.05.

**Figure 5:**
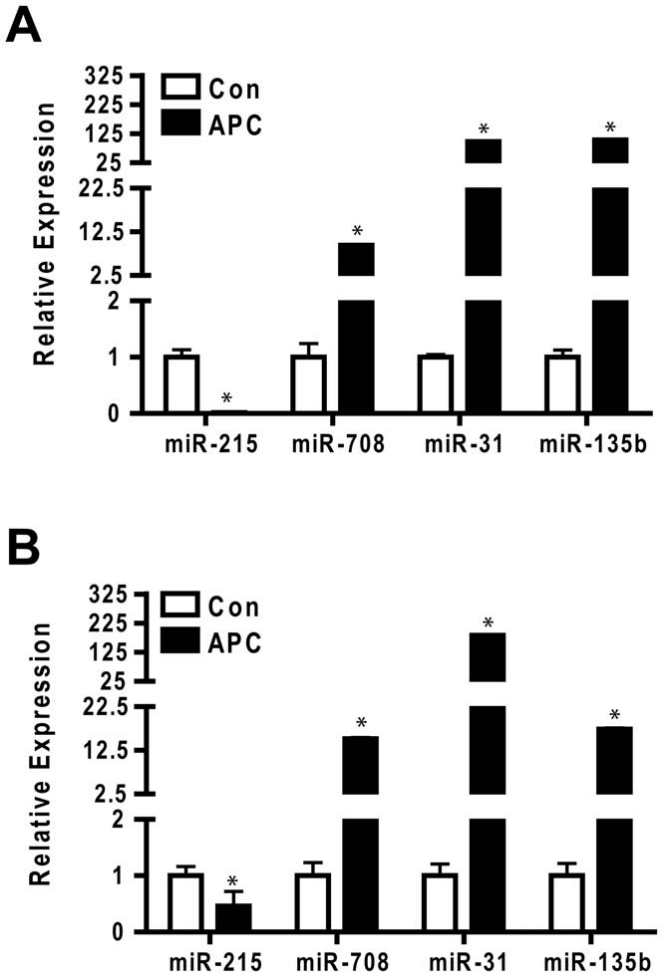
Validation of focus miRNA in an independent sample set. RNA was prepared from an independent set of APC tumors (n = 9) and controls (n = 9) (**Panel A**) or CAC tumors (n = 4) and controls (n = 8) (**Panel B**). MicroRNA abundance was measured by Taqman microRNA specific stem PCR and normalized with sno135. Data were normalized using Statminer and calibrated to expression of individual miRNAs in the control samples. Bars represent mean and standard deviation. * indicates p-value<0.05.

We used conventional TaqMan cDNA conversion primers and qPCR reagents to confirm differential expression of the four miRNAs that exhibited common regulatory responses in the CAC and APC tumor samples used in the initial multiplex qPCR analysis. As shown in Figure 4, repression of miR-215 was confirmed in both CAC and APC tumor samples, whereas induction of miR-708, miR-31, and miR-135b was likewise confirmed in tumors of both origins. We prepared an additional set of controls and tumors from both chronic colitis and APC mice and measured the abundance of miR-215, miR-708, miR-31, and miR-135b. Figure 5 shows that within experimental error the results predicted from the rank order statistical analyses were validated in an independent sample cohort.

### Focus miRNA expression in primary human colon cancers

The pattern of expression of the 8 focus miRNAs listed in Table 1 implies that some or all of these miRNAs may play a potential role in the etiology of the early stage adenomas that form in APC^Min/+^ mice and/or mice that have undergone experimental chronic colitis. A literature survey indicates that some of these focus miRNAs have been previously reported to be differentially expressed in primary human colon cancers, as shown in Table 2. MicroRNAs miR-31 and miR-135b have been reported to be induced in colon cancer, similar to the response that we have observed in early stage tumors in chronically inflamed or APC^Min/+^ mice. Likewise, we observed downregulation of miR-215 in tumors of both types, and downregulation of miR-133a in CAC but not APC tumors. Both of these miRNAs are reported to be downregulated in human colon cancers [18], [21]–[23], [37]. Our analyses indicate that these miRNA species are induced very early in transformation and probably affect signaling pathways that are central to processes that are independent of the mechanism of initiation.

**Table 2:** Tumor expression levels and validated target genes/functions of the eight focus miRNAs.

miRNA	Tumor Level	References	Validated Targets	Validated Biological Functions	References
miR-31	up	[12]–[17], [22], [23], [25], [39]	TIAM1	migration, invasion	[42]
miR-135b	up	[13]–[15], [20], [27], [33]	APC		[26]
miR-137	down	[13], [15], [21], [28]	CDC42	cell cycle, invasion	[29]
miR-133a	down	[12], [15], [21]			
miR-215	down	[18], [21]–[23], [37]	TYMS, DTL, DHFR	cell cycle, adhesion, proliferation, chemoresistance	[30], [31], [35], [44], [45]
miR-708	not reported				
miR-467d	n.a.*				
miR-218	not reported				

Expression of microRNAs in human colon tumors compared to normal controls as indicated in corresponding references.

*For miR-467d, no human homolog is known. Differential expression of miR-708 and miR-218 was not reported in the literature. Validated gene targets and biological functions of individual miRNAs are indicated with corresponding references. TIAM, t-cell lymphoma invasion and metastasis 1. APC, adenomatosis polyposis coli. TYMS, thymidylate synthetase. DTL, denticleless protein homolog. DHFR, dihydrofolatereductase. CDC42, cell division cycle 42.

### Analysis of potential miRNA targets and functions in early stage tumors

Several different algorithms have been developed to predict miRNA targets, based upon different parameters to assess complementarily of miRNA seed sequences to sequences within the 3′ untranslated regions of mRNAs [10], [57]–[59]. Although these analyses are far from perfect in terms of predictive ability, they remain the only tools available for predicting miRNA targets, and the output of these analyses is useful for predicting hypothetical connections between miRNAs, targeted pathways, and biological functions. Since different algorithms often yield different results, we used PicTar, MicroCosm, TargetScan, and DianaT to generate lists of potential targets for each of the differentially expressed miRNAs. We included in these lists only targets that were conserved in mouse and human transcripts, and the individual predictions were pooled to form a master list of putative targets for all four miRNAs. This list was then collated with microarray data from isolated mouse distal colonic epithelial cells [60], and targets that were not scored as ‘Present’ in these cells were eliminated. The curated lists were then used for pathway prediction.

We focused on the 4 miRNAs that were differentially expressed in early stage lesions of both genetic and inflammatory origin (miR-215, miR-31, miR-708, miR-135b). Using the filters described above, we identified 527 potential target mRNAs that were expressed in mouse distal colonic epithelial cells. Gene ontology analysis (Table 3) identified Cancer as the top biological function associated with this gene set, with 130 of the 527 targets linked to Cancer through GO terms (p-value range 5.86E^−05^ to 2.87E^−02^). GO terms associated with Gene Expression emerged as the top Molecular and Cellular Function, with 131 targets linked to this category (p-value range 5.78E^−11^ to 2.87E^−02^). The top Tox function was TGF-β Signaling (p = 5.2E^−04^, with 9/77 signaling components identified as potential targets). The remaining statistically significant Tox functions were related to nuclear receptors that have been studied extensively within the context of colonic epithelial cell physiology and pathology, including TR/RXR, VDR/RXR, and FXR/RXR.

**Table 3:** Summary of top GO functions identified for miR-215, miR-31, miR-135b, and miR-708 by Ingenuity Pathway Analysis.

GO Functions	p-value	# of Genes
***Top Biological Function-Disease***		
Cancer	5.86E^−05^-2.87E^−02^	130
***Top Biological Function-Molecular and Cellular Function***		
Gene Expression	5.78E^−11^-2.87E^−02^	131
***Canonical Pathways***		
Molecular Mechanisms of Cancer	1.14E^−06^	28/372
***Tox Lists***		
TGF-β Signaling	5.3E^−04^	9/77
TR/RXR Activation	3.63E^−03^	8/83
VDR/RXR Activation	8.73E^−03^	7/77
FXR/RXR Activation	1.29E^−02^	7/83
G1/S Transition of the Cell Cycle	1.60E^−02^	5/49

**Figure 6:**
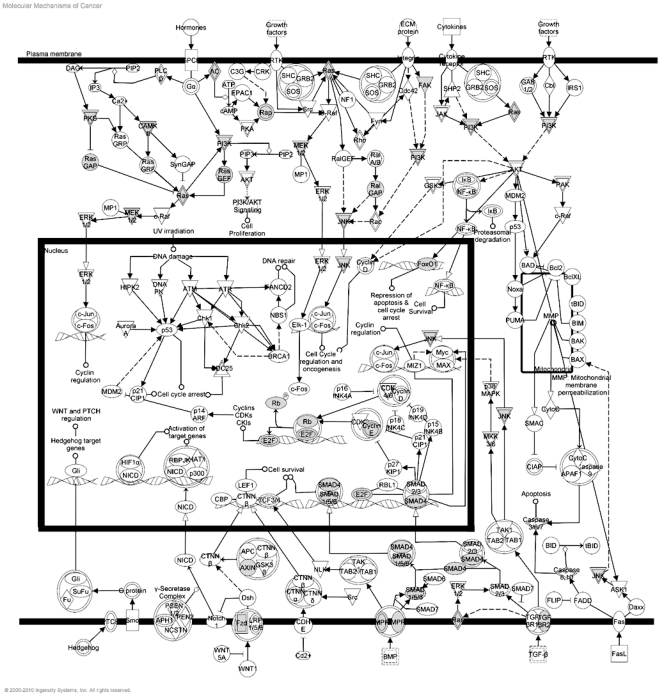
Focus miRNAs are predicted to regulate several key nodal signaling events that are known to be involved in transformation. Putative mRNA targets for the 4 focus miRNAs were compiled using PicTar, MicroCosm, TargetScan, and DianaT databases. Predicted targets were overlaid in gray on the Ingenuity Molecular Mechanisms of Cancer canonical pathway.

These hypothetical GO functions predict a strong linkage between the four differentially expressed miRNAs and transformation of colonic epithelial cells. This hypothetical linkage is emphasized by the data shown in Figure 6, in which putative miRNA targets (shown in green) are overlaid onto the knowledge-based (Ingenuity) Molecular Mechanisms of Cancer canonical pathway (p = 1.14E^−06^, 28 predicted targets/372 pathway components). Critical membrane signaling functions related to Ras/MAPK, PI3K, and WNT/β-catenin represent potential mechanistic links between these miRNAs and transformation, whereas nuclear functions linked to RB/E2F control emerge as potential mediators of cell cycle control. Finally, as indicated by the GO analysis of Tox function, TGF-β/BMP/SMAD signaling is nominated as a very significant potential link between these four miRNAs and transformation of colonic epithelial cells.

## Discussion

Our central objective was to compare miRNA expression in colon tumors that arise due to chronic inflammation and germ line mutations in model systems. It was clear early on that normalization of the data would be an issue, since we were comparing very different tissue states (*e.g.*, chronically inflamed mice on standard lab chow versus aged breeders on high fat diets); and there are no well-established objective criteria for identifying appropriate endogenous controls for normalization of miRNA expression under such conditions (For a review of this issue, see [61]). Our first objective was therefore to use an independent sample set to compare different models for normalization. To this end we prepared and analyzed distal colonic epithelial cells from control and acutely inflamed tissues, using a conventional DSS acute colitis model. We examined an early, pre-inflamed, state, prior to detectable loss of histological integrity of the distal colonic epithelium, thinking to minimize changes in miRNA expression that might be associated with massive infiltration of immune cells. Using three different analytical approaches we identified 51 high priority miRNA targets that are likely to play a significant role in controlling gene expression in epithelial cells in the very earliest stages of DSS-induced inflammation. Although these findings represent, to our knowledge, the first detailed report of differential miRNA expression in this colitis model, we have not further analyzed these data. Rather we used these data to validate our choice of rank order statistics to identify differentially expressed miRNAs. This analytical approach does not require identification of appropriate internal controls and is particularly appropriate for analysis of relatively small groups of samples, since no assumptions are required about equal RNA loading or efficiency of extraction of miRNAs or cDNA conversion from very different kinds of tissues [62]. In general, the rank order differences that we observed correlate well with analog miRNA abundance, expressed as DCt in the median normalized data or DDCt in the sno135/miR-25 normalized (Statminer) analyses. However, the p-values assigned by the different statistical models varied considerably, due at least in part to the small sample size and the use of moderated (Limma empirical Bayesian) versus unmoderated (t-test) approaches to estimating variance. Given that any statistical model is likely to break down with small sample sets, we elected to use the simplest, most conservative approach: rank order statistics. We used Statminer normalization (against sno135 and miR-25) to extract relative miRNA abundance.

Rank order statistics was used to identify 8 differentially expressed miRNAs in tumors that formed in the distal colons of APC^Min/+^ mice and chronically inflamed mice. It should be noted that these tumors are primarily adenomas and thus represent a very early stage in transformation. We should also emphasize that these 8 miRNAs represent only the most prominent, and therefore likely the most reproducible, changes in miRNA expression in these samples. Many other miRNAs exhibited statistically significant changes in rank order (as well as normalized Ct values) in one or another of these sample sets, but our confidence in these targets is limited by the small sample size. We have not undertaken to expand our sample size, given the cost of the TaqMan arrays and the fact that it takes over 40 weeks to generate CAC tumors. Nevertheless, we were able to validate our four most prominent miRNAs (miR-215, miR-708, miR-135b, miR-31) in an independent set of APC and CAC tumors. These four miRNAs are therefore firmly established as differentially expressed in early stage tumors of both genetic and inflammatory origin.

Target identification remains the greatest challenge to determining the functional significance of differential miRNA expression. We have attempted to be as conservative as possible in target prediction, using an approach that combines predicted targets from four of the most popular target prediction programs and curating these predicted targets to include only those that are conserved in mouse and human and are expressed in distal colonic epithelial cells. Our analysis suggests that these four miRNAs, in the aggregate, may affect signaling through a number of important pathways that have been linked to colon carcinogenesis. Notable among these are MAPK, PI3K, WNT, and TGF-β signaling, all of which are well-known to be intimately involved in transformation of colonic epithelial cells. The precise links between any or all of these pathways and individual miRNAs remains to be established. Nevertheless, the observation that these miRNAs are coordinately dysregulated in tumors of very different origins in combination with the hypothetical connections between these miRNAs and known oncogenic signaling pathways strongly suggests that this group of miRNAs plays some critical role or roles in the very earliest stages of transformation of colonic epithelial cells.

One obvious question relates to the link between these focus miRNAs and inflammation. The observation that these miRNAs are differentially expressed in APC tumors indicates that their identification in the CAC tumors is not simply a reflection of some residual inflammatory state that persists in the colonic epithelium of CC mice. Moreover, most of these miRNAs are only modestly dysregulated in the AC samples, on the order of 2- to 4-fold which may not be significant in these samples. MiR-31 is not induced in AC samples and is clearly not related to inflammation by that criterion. There have been a limited number of studies on miRNA expression in human inflammatory bowel disease [55], [56], and none of our four focus miRNAs was linked to human colitis. MicroRNA expression in human colon tumors has been investigated in some detail. Interpretation of these data is confounded by the fact that there is generally little overlap between the data reported by different groups, using different analytical platforms, different sample cohorts, and different analytical pipelines. Nevertheless, there is evidence that miR-31 [12]–[17], , miR-135b [13]–[15], [20], [21], [33], and miR-215 [18], [21]–[23], [37] are differentially expressed in fully transformed colonic epithelial cells. In addition, a recent publication by Olaru indicates that miR-31 and miR-21 are also upregulated in human colitis-associated neoplasia [25], supporting our data that suggests a role of these miRNAs in transformation of non-IBD and IBD-associated cancer. Of note, our data also indicates that miR-215 is downregulated in APC and CAC tumors and in human colon tumors [18], [21]–[23], [37]. In contrast, Olaru et al. observed an upregulation of miR-215 in colitis-associated neoplasia [25]. The cause of this discrepancy is unclear, but is tempting to speculate the differential expression of miR-215 is reflected by the stage of transformation, dysplasia versus carcinoma. We also note that currently there is no report of miR-708 in human colon cancers, in contrast to our data which indicates that this miRNA is dramatically induced in mouse colonic tumors. Likewise, miR-218 is induced in colitis-associated tumors but is not induced in APC tumors and has not been reported to be induced in human tumors. It is tempting to speculate that miR-218 may play some role that is specific to progression from inflammation to transformation in the colon, but this hypothesis remains to be evaluated. Overall, our analyses indicate that these four focus miRNAs are dysregulated very early in transformation and the observation that these changes appear to be independent of the mechanism of tumorigenesis (genetic versus inflammatory) suggests that these species have some fundamental function(s) that is required for initiation of transformation. The challenge now is to elucidate those functions so as to identify early events that can be manipulated for therapeutic or preventive intervention.

## Materials and Methods

### Ethics Statement

All mice were maintained as part of an American Association for Accreditation of Laboratory Animal Care facility. Animal experimentation was conducted in accordance with accepted standards of humane animal care according to the protocol #A17008 approved by the Mayo Clinic College of Medicine Institutional Animal Care and Use Committee Animal.

### Animal studies

Acute and chronic dextran sodium sulfate (DSS) treatment protocols were initiated on six week old female C57BL/6J mice (Jackson Labs) maintained on standard AIN-76A rodent diet. Acute DSS colitis (abbreviated AC) was induced by administering 3.5% dextran sodium sulfate (M.W. 36,000–50,000, MP Biomedicals) *ad libitum* in filter-purified drinking water for four days according to the well-established procedure of Okayasu et al. [63]. To induce chronic colitis (CC) and colitis-associated tumors (CAC), mice were subjected to 12 cycles of inflammation followed by recovery, to mimic recurrent bouts of colitis in human patients [63], [64]. Each cycle consisted of 1% DSS in drinking water for seven days followed by a 10 day recovery (normal water). APC^Min/+^ mice were retired breeders, approximately 120 days of age and maintained on a high fat diet (Breeders Chow).

Mice were sacrificed and colons were dissected and flushed with cold phosphate-buffered saline. Each colon was opened longitudinally to expose the luminal surface and fixed flat in 10% formalin for 12 hr. Tissues were washed three times with colon 1× PBS and stored in 70% ethanol at 4°C. All analyses were performed using tissue from the distal colon since this is the site of tumor formation in both CAC and APC models. A 1 cm section of epithelium was scraped 1 cm from the distal end of colons from mice on the acute colitis, chronic colitis, and control protocols. Some animals that underwent the chronic colitis protocol developed tumors in the distal colon, whereas almost all APC^Min/+^ mice had 1–2 distal colon tumors. Tumors were dissected for RNA extraction. For APC^Min/+^ mice, epithelial scrapings adjacent to isolated distal tumors served as controls. Total number of samples analyzed for each condition were: 4 normal epithelium, 4 acute colitis, 4 chronic colitis, 4 colitis-associated tumors, 4 APC^Min/+^ control epithelium, and 7 APC^Min/+^ tumors. An additional validation set of APC tumors (n = 9) and controls (n = 9) or CAC tumors (n = 4) and controls (n = 8) was also prepared.

### RNA isolation and qRT-PCR

Total RNA was extracted with the Recover All™ Total Nucleic Acid isolation kit as described by the manufacturer (Ambion). The abundance of individual miRNAs was determined using conventional two-step quantitative reverse transcriptase-mediated real-time PCR (qPCR). 10 ng of total RNA was converted to cDNA with Taqman mirRNA RT transcription kit and primer specific probes. MicroRNA qPCR reactions were performed in triplicate with 10 ng cDNA and the TaqMan Universal no UMG PCR master mix. All primers and probes were purchased from Applied Biosystems. All amplification data were collected with an Applied Biosystems Prism 7900 FAST sequence detector and analyzed with Sequence Detection System software (SDS ver. 2.3, Applied Biosystems).

### TaqMan miRNA low density array

The Taqman© Rodent MicroRNA Array A v2.0 was used to assess miRNA expression in RNA samples from epithelium of normal mouse colon, acute DSS colitis, chronic DSS colitis, chronic colitis tumors, and of APC^Min/+^ tumors and adjacent tissue (see animal studies). Briefly, 500 ng of total RNA was reverse transcribed with Megaplex RT primers and Taqman MicroRNA RT kit. Samples were then amplified with Megaplex Preamp primers and Taqman Preamp master mix. cDNAs were diluted 1∶4.33 and loaded with Taqman universal PCR master mix on each low density array according to manufacturer's instructions. Amplification kinetics were measured on an Applied Biosystems Prism 7900 FAST sequence detector and analyzed with Sequence Detection System software (Applied Biosystems).

### Statistical analysis

Stats Direct (ver. 2.6.1) was called from Excel 2007 to carry out t-test and regression analyses. Limma was run in an R environment (ver. 2.4.1) and called from Statminer ver. 4.2 (Integromics, Inc.) to calculate hyperparameters that were then used to moderate variance estimates in a variation of the t-test. Statminer uses this empirical Bayesian approach to estimate variance (defined as M function using the Genorm default) among user-defined endogenous controls. We selected sno135 and miR-25 as normalizers since both had M functions <0.15 individually and a combined M function of <0.1.

### miRNA target prediction

Potential miR targets were determined by combining the results from TargetScanv.5.1 (http://www.targetscan.org), MicroCosm v5 (http://www.ebi.ac.uk/enright-srv/microcosm/tdocs/targets/v5/), DIANA-microTv3.0 (http://diana.cslab.ece.ntua.gr/microT/) and Pictar (http://pictar.mdc-berlin.de/). Potential target sequences were pooled for both mouse and human mRNAs and only those targets that were conserved between the two species were retained. Finally, we aligned these predicted targets against our published Affymetrix gene expression profiles from mouse distal colonic epithelial cells [60]. Predicted targets that were not scored as ‘Present’ in this dataset were eliminated from further analysis. Networks were generated through the use of Ingenuity Pathway Analysis (Ingenuity Systems®, www.ingenuity.com). Functional analysis was used to identify the biological functions and/or diseases that were most significant to the molecules in the network. The network molecules associated with biological functions and/or diseases in Ingenuity's Knowledge Base were considered for the analysis. Right-tailed Fisher's exact test was used to calculate p-value determining the probability that each biological function and/or disease assigned to that network is due to chance alone.

## Supporting Information

Table S1 **Expression values of differentially expressed miRNAs in DSS-induced acute colitis as determined by three different normalization method.** Shown are differentially expressed miRNAs scored as significantly different (P≤0.01) in one or more of the analyses: rank order statistics (Rnk_Diff), t-test using median normalized data (Med_Norm), or the Statminer implementation of Limma (Limma_SM). Using additional filters of ≥(±10),P≤0.01 for rank difference and ≥(±1.0) P≤0.01 for Med_Norm and Limma_SM approaches, 51 miRNAs (shown in bold) overlapped among the three normalization methods.
(XLSX)

Table S2 **Expression values of miRNAs in CAC and APC tumor samples.** Raw Ct values for miRNAs are shown for CAC (n = 4) and APC (n = 5) tumor samples. Detectors that were scored as ‘undetectable’ were assigned Ct values of 40. Additionally, rank order statistics (Rnk_Diff) was used to compare miRNA profiles in CAC versus APC tumors. Each miRNA within a given sample was force ranked on analog readout of miRNA abundance (Ct value). All miRNAs with Ct = 40 were assigned the same rank within a sample. A t-test (assuming unequal variance) was then carried out to compare the rank order assignments of miRNAs in the two different groups so as to identify miRNAs whose mean rank order of expression changed significantly.
(XLSX)
